# Supplementary material for: COVIDReady2 study protocol: cross-sectional survey of medical student volunteering and education during the COVID-19 pandemic in the United Kingdom
Source: BMC Med Educ. 2021 Apr 14;21:211. doi: 10.1186/s12909-021-02629-4 (PMC8045566; doi:10.1186/s12909-021-02629-4)
Supplement: Supplementary file 1 — Additional file 1. Appenfix B. Survey. *Compulsory (only three questions). [file 12909_2021_2629_MOESM1_ESM.docx]

**APPENDIX B**

**SURVEY**

*Compulsory (only three questions)

| **Question** | **Answer options** |
| --- | --- |
| **1. Demographics** |  |
| Did you volunteer or work in a clinical capacity during the pandemic?* | Yes/No |
| Year at medical school when the COVID-19 pandemic started (not counting any intercalated years)* | DROP DOWN 1-5, intercalation year 3, intercalation year 4 |
| Are you on a four-year graduate entry medical course?* | Yes/No |
| Medical school | DROP DOWN of all UK medical schools & prefer not to say |
| Age (at time of volunteering) | NUMERICAL, limit 16-70 years old |
| Gender | MULTIPLE CHOICE Male, female, non-binary, prefer not to say, other [free text] |
| Ethnicity | MULTIPLE CHOICE  White - British  White - Irish  White - Gypsy or Irish Traveller  White - Any other white background  Mixed/Multiple ethnic groups - White and Black Caribbean  Mixed/Multiple ethnic groups - White and Black African  Mixed/Multiple ethnic groups - White and Asian  Mixed/Multiple ethnic groups - Any other mixed/multiple ethnic background  Asian/Asian British - Indian  Asian/Asian British - Pakistani  Asian/Asian British - Bangladeshi  Asian/Asian British - Chinese  Asian/Asian British - Any other Asian/Asian British Background  Black/African/Caribbean/Black British - African  Black/African/Caribbean/Black British - Carribean  Black/African/Caribbean/Black British - Any other Black/African/Caribbean/Black British  Other ethnic group - Arab  OTHER (FREE TEXT)  Prefer not to say |
| **QUESTIONS ONLY IF THEY VOLUNTEERED** |  |
| **Volunteer role** |  |
| Which deanery did you volunteer in? | DROP DOWN  East Anglia  Essex, Bedfordshire & Hertfordshire  Leicester, Northamptonshire & Rutland  London & KSS: (North Central and East London; North West London; South Thames)  North West of England  Northern  Northern Ireland  Oxford  Peninsula  Scotland  Severn  Trent  Wales  Wessex  West Midlands (Central, North & South)  Yorkshire & Humber |
| Is your medical school in this deanery? | Yes/No |
| How many miles away from your home did your volunteering role take place? | 0  1-10  11-20  21-30  31-40  41-50  50+ |
| When did you start volunteering or working? | DATE |
| How many weeks did you volunteer or work for? | NUMERICAL VALUE |
| Which months did you volunteer in? | CHECKBOX  March 2020  April 2020  May 2020  June 2020  July 2020  August 2020  September 2020  October 2020  November 2020  December 2020  January 2021  February 2021  March 2021  April 2021  May 2021 |
| On average how many hours a week did you volunteer or work for? | MULTIPLE CHOICE  0  1-5  6-10  11-15  16-20  21-25  26-30  31-35  36-40  41-45  46-50  50+ |
| Was this a paid role? | Yes/No |
| Where did you volunteer or work? | Hospital  General Practice  OTHER (FREE TEXT) |
| What was the main role you had when volunteering or working? | MULTIPLE CHOICE  Administrative role (e.g. ward clerk, secretary, receptionist, 111 caller)  Allied healthcare professional/technician  Carer/care assistant (in hospital or care home)  Dentist  Doctor (interim foundation or equivalent)  First responder/first aider  Healthcare assistant  Healthcare scientist (e.g. hospital laboratory, clinical or laboratory research)  Nurse  Paramedic/emergency medical technician  Pharmacist  Pharmacy assistant  Phlebotomist  Doctor’s assistant  Patient liaison  Primary care (General Practice) role (e,g: summariser, Health Care Assistant, Quality Improvement/Audit)  Vaccinator  OTHER (FREE TEXT) |
| Did you hold this role prior to the pandemic? | Yes/No |
| Please describe what this role involved | FREE TEXT |
| **Opinions** |  |
| How did volunteering benefit your learning? (both as a medical student and preparation to practice as a Foundation Year 1 doctor) | FREE TEXT |
| How did volunteering disadvantage your learning? (both as a medical student and preparation to practice as a Foundation Year 1 doctor) | FREE TEXT |
| I would be willing to perform the same role in a non-pandemic setting as a formal part of my medical degree programme | LIKERT |
| I found my role as a volunteer more useful for my learning, than my role as a medical student on a clinical placement in a similar setting | LIKERT |
| Why did you find the role as a volunteer more or less useful than your role as a medical student on a clinical placement in a similar setting? | FREE TEXT |
| How has your volunteering experience affected your approach to patients? | FREE TEXT |
| Has your volunteering experience changed your view of how the NHS or other healthcare facilities work and if so how? | FREE TEXT |
| Were any opportunities provided for reflective practice? | Yes/No |
| IF YES TO REFLECTIVE PRACTICE |  |
| What opportunities were made for reflective practice? | CHECKBOX  Portfolio logs  Diaries  In-person reflective discussions  OTHER (FREE TEXT) |
| I found the reflective practice provisions useful. | LIKERT |
| IF NO TO REFLECTIVE PRACTICE |  |
| I think reflective practice provisions would have been useful. | LIKERT |
| **Issues** |  |
| What issues did you encounter while volunteering during the coronavirus pandemic? | FREE TEXT |
| Did you do on-calls? | MULTIPLE CHOICE  None  Day  Night  Day and Night |
| Did you work with patients who were COVID-19 positive? | Yes/No |
| I understood my role and the specific tasks I was expected to undertake | LIKERT |
| I felt competent to perform my role. | LIKERT |
| I felt other staff were aware of my role and the specific tasks I was expected to undertake | LIKERT |
| I was asked to perform skills outside my competence. | LIKERT |
| I felt confident saying no to tasks I was not adequately prepared for. | LIKERT |
| I felt patient safety was compromised while volunteering. | LIKERT |
| I received adequate supervision at work. | LIKERT |
| Did you have a named supervisor? | MULTIPLE CHOICE  Yes - clinical and educational supervisor  Yes - clinical supervisor only  Yes - educational supervisor only  No |
| I received a contract | MULTIPLE CHOICE  Yes - prior to work  Yes - after starting work  No |
| I was told who I could speak to if I had personal, health, or mental health problems while at work | Yes/No |
| What additional training did you receive for your role? (Please tick all that apply) | CHECKLIST  None  Induction  How to report clinical incidents (e.g. Datix)  First aid training  Basic life support training  Basic infection control training (e.g. handwashing)  Surgical scrubbing and gowning  Infection control training with personal protective equipment (e.g. respirator masks, donning and doffing of goggles and garments)  Pandemic influenza teaching  OTHER (FREE TEXT) |
| Did you test positive for COVID-19 while volunteering? | Yes - swab test positive  Yes - swab test negative, but confirmed by health professional  Yes - suspected due to symptoms (e.g. fever, cough, shortness of breath, loss of smell)  No |
| **QUESTIONS ONLY IF THEY DIDN’T VOLUNTEER** |  |
| What were reasons that you did not volunteer? | FREE TEXT |
| Have you had any negative experiences by not volunteering? | Yes/No |
| If you did have negative experiences by not volunteering what were they? | FREE TEXT |
| **QUESTIONS FOR ALL** |  |
| I would be willing to volunteer to work in the future if the need arose. | LIKERT |
| I believe the period from the start of the first lockdown (23 March 2020) until now has benefited my medical education / career. | LIKERT |
| I believe the period during the first lockdown (23 March 2020 to 4 July 2020) has benefited my medical education / career. | LIKERT |
| The skills I have developed during the first lockdown (23 March 2020 to 4 July 2020) have prepared me for FY1 | LIKERT |
| The skills I have developed during the first lockdown (23 March 2020 to 4 July 2020) have prepared me for the 2020-2021 academic year | LIKERT |
| How have these skills prepared you for FY1 or the 2020-2021 academic year? | FREE TEXT |
| I feel assistantships (final year clinical placements) should be replaced with formal roles within a clinical team | LIKERT |
| What are your thoughts on replacing assistantships (final year clinical placements) with formal roles within a team (paid or unpaid)? | FREE TEXT |
| How many times did you perform the following skills during the first lockdown (23 March 2020 to 4 July 2020): | Venepuncture  Cannulation  Prescription of medications  History or examination of patient  Organise investigations for patients (e.g. blood tests, x rays etc)  Formulate management plans  Prescribe fluids  Measure observations/vital signs  Communicate information to patients  Communicate information to other healthcare professionals  Administrative medical duties (e.g. documentation, discharge letters etc)  Complete a chronic disease review  Communicate with patients as part of a medication review or audit    MULTIPLE CHOICE for each skill  None  Observed  Assisted  Performed 1-5 times (independently)  Performed 6-10 times (independently)  Performed 10+ times (independently) |
| What support did you receive from your medical school to facilitate volunteering? | FREE TEXT |
| Follow up study |  |
| Please enter your email address if you would be willing to be contacted for a future study.    We may conduct a follow up study to understand in greater depth responses from this study. This would either be by a focus group discussion with a researcher and other survey participants, by a semi-structured interview with a researcher, or an additional survey. By entering your email address it does not commit you to participating in a future study.    Your email address will be separated from the data entered before the data is analysed, will not be shared outside the data collection team, and will be deleted in two years time. | EMAIL |
